# The physiological and biochemical basis of potency thresholds modeled using human estrogen receptor alpha: implications for identifying endocrine disruptors

**DOI:** 10.1007/s00204-024-03723-4

**Published:** 2024-05-05

**Authors:** Christopher J. Borgert, Lyle D. Burgoon, John C. Matthews

**Affiliations:** 1 Applied Pharmacology and Toxicology, Inc. and CEHT, Univ. FL College of Vet. Med., Gainesville, FL USA; 2 Raptor Pharm & Tox, Ltd, Apex, NC USA; 3 University of Mississippi School of Pharmacy, University, MS USA

**Keywords:** Affinity, Dietary supplements, Efficacy, Endocrine active substances, Endocrine disruption, Endocrine pharmacology, Environmental estrogen, Hormone, Intrinsic activity, Potency, Potency threshold, Receptor occupancy

## Abstract

The endocrine system functions by interactions between ligands and receptors. Ligands exhibit potency for binding to and interacting with receptors. Potency is the product of affinity and efficacy. Potency and physiological concentration determine the ability of a ligand to produce physiological effects. The kinetic behavior of ligand-receptor interactions conforms to the laws of mass action. The laws of mass action define the relationship between the affinity of a ligand and the fraction of cognate receptors that it occupies at any physiological concentration. We previously identified the minimum ligand potency required to produce clinically observable estrogenic agonist effects via the human estrogen receptor-alpha (ERα). By examining data on botanical estrogens and dietary supplements, we demonstrated that ERα ligands with potency lower than one one-thousandth that of the primary endogenous hormone 17β-estradiol (E2) do not produce clinically observable estrogenic effects. This allowed us to propose a Human-Relevant Potency Threshold (HRPT) for ERα ligands of 1 × 10^–4^ relative to E2. Here, we test the hypothesis that the HRPT for ERα arises from the receptor occupancy by the normal metabolic milieu of endogenous ERα ligands. The metabolic milieu comprises precursors to hormones, metabolites of hormones, and other normal products of metabolism. We have calculated fractional receptor occupancies for ERα ligands with potencies below and above the previously established HRPT when normal circulating levels of some endogenous ERα ligands and E2 were also present. Fractional receptor occupancy calculations showed that individual ERα ligands with potencies more than tenfold higher than the HRPT can compete for occupancy at ERα against individual components of the endogenous metabolic milieu and against mixtures of those components at concentrations found naturally in human blood. Ligands with potencies less than tenfold higher than the HRPT were unable to compete successfully for ERα. These results show that the HRPT for ERα agonism (10^–4^ relative to E2) proposed previously is quite conservative and should be considered strong evidence against the potential for disruption of the estrogenic pathway. For chemicals with potency 10^–3^ of E2, the potential for estrogenic endocrine disruption must be considered equivocal and subject to the presence of corroborative evidence. Most importantly, this work demonstrates that the endogenous metabolic milieu is responsible for the observed ERα agonist HRPT, that this HRPT applies also to ERα antagonists, and it provides a compelling mechanistic explanation for the HRPT that is grounded in basic principles of molecular kinetics using well characterized properties and concentrations of endogenous components of normal metabolism.

## Introduction

The endocrine system functions via the interaction of small molecules with biological macromolecules. Small molecules such as hormones, metabolic intermediates, and transcriptional response modifiers, serve as endocrine effectors. Biological macromolecules, such as receptors, enzymes, transporters, DNA-response elements, etc. serve as endocrine effector sites. Those bimolecular interactions are governed by fundamental principles of receptor, enzyme and transport kinetics known as (1) potency, which is the product of the affinity of the effector and its efficacy at the effector site, and (2) mass action, which is a function of affinity and concentration. Potency and mass action thus determine the dose–response relationship for all endocrine-active molecules regardless of their origin, endogenous or exogenous. The laws of bimolecular interaction translate empirically to potency thresholds (reviewed in Borgert et al. 2013). Building upon this theoretical background, we recently derived a Human-Relevant Potency Threshold (HRPT) for estrogen receptor alpha (ERα)-agonist ligands by comparing chemicals with a wide range of potencies at ERα to effects observable in humans that are mediated via ERα (Borgert et al. 2018).

To substantiate the concept of an HRPT at a mechanistic level, it is necessary to consider the effects of competing ligands on receptor occupancy. There are numerous endogenous chemicals that can bind to ERα. These include hormones, precursors to hormones, metabolites of hormones, and a variety of other normal products of metabolism. When there are many different ligands present, those with low or no intrinsic activity when bound to the receptor will prevent ligands with high intrinsic activity from binding. This competition for receptor occupancy will reduce the abilities of both weak and strong agonists to produce a physiological effect. We have, therefore, employed mass action calculations to identify minimum levels of affinity required for any ligand to occupy meaningful fractions of estrogen receptors amidst the normal background milieu of endogenous ERα ligands. These analyses take into consideration the antagonistic actions of ligands with very low or no intrinsic activity. This allows us to explain the physiological and biochemical bases of potency thresholds that are necessary for exogenous ERα ligands to be identified as endocrine disruptive agents. These potency thresholds are highly influenced and dependent upon the potencies and background concentrations of the normal endogenous ERα ligands. Thus, the fundamental principles of endocrine pharmacology establish that a threshold approach to the identification of endocrine disruptive chemicals (EDCs) is not only justified but obligate.

Our general hypothesis is that the endogenous metabolic milieu creates a background occupancy at endocrine effector sites against which low-potency exogenous effectors cannot successfully compete. In this context, we define successful competition for occupancy at endocrine effector sites as a level that can displace the endogenous effector (e.g., endogenous hormone, enzyme substrate, or response modifier) to an extent greater than does the rest of the endogenous metabolic milieu across its normal physiological concentration range. Therefore, the background equilibrium receptor occupancy by the endogenous metabolic milieu provides a mechanistic explanation for the HRPT observed for agonist activity via ERα through empirical evaluation of clinical, rodent, and in vitro data (Borgert et al. 2018).

To conduct a specific test of this hypothesis, we calculated fractional receptor occupancies for ERα ligands with potencies below and above the previously established HRPT when normal circulating levels of some endogenous ERα ligands and the primary endogenous estrogen, E2 were also present. The general hypothesis predicts that sub-HRPT ligands (i.e., ligands with potencies less than the minimum necessary to produce physiologically observable effects via ERα agonist pathway) are unable to compete successfully for occupancy at ERα amidst the endogenous metabolic milieu. Successful competition for occupancy at ERα is defined as displacement of the endogenous hormone E2 to an extent greater than that of the endogenous metabolic milieu across its normal physiological concentration range.

Fractional receptor occupancy calculations show how successfully individual ligands with potencies above and below the HRPT can compete for occupancy at ERα against individual components of the endogenous metabolic milieu and against mixtures of these components at concentrations found naturally in human blood. Unsuccessful competition of a sub-HRPT ligand with endogenous ERα ligands, as measured by fractional receptor occupancy, would support our proposition that competition with the endogenous metabolic milieu provides a physiological basis for our observed HRPT for ERα agonism of 1 × 10^–4^ relative to the endogenous hormone E2. The hypothesis would be refuted if a sub-HRPT ligand can achieve a successful level of receptor occupancy in the presence of physiological concentrations of the endogenous metabolic milieu and the endogenous hormone E2. Unsuccessful competition at ERα by a supra-HPRT ligand amidst the endogenous milieu would suggest that our published HRPT is too low to accurately distinguish potentially endocrine active chemicals from those that lack such potential.

## Pharmacological background

This section explains the underlying pharmacological principles used to test the proposed hypotheses and may be useful for readers seeking more detail regarding their theoretical and empirical basis. The endocrine system functions by interactions between ligands and receptors. Ligands are small molecules. These include hormones and hormone-like substances. Receptors are biological macromolecules that function as endocrine effector sites. These may include receptors, enzymes, transporters, DNA-response elements, etc. Although we will use estrogen receptor ligands for the present explanation and analysis, it is important to understand that these same principles apply to ligands for all functional biomacromolecules, such as receptors, enzymes, transporters, ion channels, DNA response elements, etc. (Borgert et al. 2013; 2018; Matthews 1993).

Receptors have binding sites on and in their surfaces in which a ligand will fit with high degrees of specificity for size, shape, and chemical properties to form non-covalent, reversible binding interactions. Molecules are constantly in motion. This results in collisions between them. If there is an attractive force between a receptor and a ligand they can bind together. This attractive force is affinity. Affinity is a function of the relative rates of the molecules coming together and coming apart again. This is described by the reaction L + R ↔ LR. Increasing the ligand concentration will drive the equilibrium condition to the right while decreasing the ligand concentration drives the equilibrium condition to the left. The equilibrium condition is described by the mass action equation, Eq. 1.

1 $$Kd =\frac{\left[L\right]\left[R\right]}{\left[{\text{LR}}\right]}$$

At equilibrium [*L*] is the concentration of unbound ligand, [*R*] is the amount of unbound receptor, and [LR] is the amount in complex. [LR] is also the fractional receptor occupancy.

The equilibrium dissociation constant (*K*_d_) is the measure of the strength of the interaction, i.e., the affinity of *R* and *L* for one another. *K*_d_ has the units of concentration (moles/liter). When [LR] is large relative to [*L*][*R*], *K*_d_ will be small. The smaller the value of *K*_d_ the higher the affinity. Rearranging the mass action equation gives us the Langmuir binding isotherm (Langmuir 1918), Eq. 2.

2 $$\left[{\text{LR}}\right]=\frac{\left[L\right]\left[{\text{LRmax}}\right]}{Kd+\left[L\right]}$$

[LRmax] is when 100% of the receptors are occupied by the ligand. When [*L*] is 1/100th of *K*_d_ the fractional receptor occupancy is a little less than 1% (0.9901%). When [*L*] is 1/1000th of *K*_d_ the fractional receptor occupancy is a little less than 0.1% (0.09901%).

Binding alone is not enough to explain endocrine system action. To produce an effect the ligand must have intrinsic activity; it must be able to stimulate the receptor by converting the receptor into a state in which it can produce a physiological effect. Ligands that have intrinsic activity are termed agonists. Not all ligands have the same level of intrinsic activity. Some ligands can bind to the receptor without being able to stimulate the receptor. These ligands would occupy the binding site and when bound would prevent other ligands that have intrinsic activity from being able to access the binding site. These ligands are termed antagonists. Other ligands may be able to stimulate the receptor, but they may not be able to effectively stabilize the stimulated state resulting in reduced ability to produce the physiological effect. These ligands are termed partial agonists or weak agonists. The relative ability to produce the physiological effect is termed efficacy. Potency is the product of affinity and efficacy.

These laws of bimolecular interaction translate empirically to potency thresholds (reviewed in Borgert et al. 2013). As explained therein, the biological milieu of molecules capable of interacting with various receptors is critical for understanding whether exogenous chemicals may produce effects via the endocrine system. Functionally active hormones are present in the extracellular milieu at concentrations in the range of 10^–11^ to 10^–9^ molar amidst a 10^6^- to 10^9^- fold molar excess of structurally similar, non-hormone molecules, such as sterols, amino acids, peptides, as well as hormone precursors and metabolites (Chedrese and Celuch 2009; Grannar 1993). This presents formidable challenges to maintaining a functional and efficient hormone-based communication system wherein target cells must clearly distinguish molecules that convey critical physiological information from structurally similar molecules in the body that do not.

Cells that receive endocrine signals achieve this distinction by selective binding interactions between hormones and their cognate receptors. These binding interactions are highly specific for size, shape, and chemical properties. Only those pairings that also produce conformational alterations in the receptors to achieve the stimulated state can produce biological signals (Chedrese 2009; Chedrese and Celuch 2009). The effectiveness of these pairings is reflected by potency, a property of bimolecular interactions comprised of affinity and efficacy. Affinity and efficacy are interdependent conceptual entities developed by empirical observation over decades in pharmacology to explain lateral and horizontal shifts in dose–response phenomena at the molecular mechanistic level (reviewed in Colquhoun 2006; Kenakin 2004, 2009; Negus 2006; Rang 2006). Affinity relates to the strength of attraction between the ligand and its receptor, and efficacy relates to the ability of the bound ligand to alter the functional state of the receptor. Affinity determines the fraction of receptors of a particular type that will be bound by a particular ligand at any specific ligand concentration, and efficacy determines the functional result of that occupancy. High potency is the property that allows hormones and other signaling molecules to alter the functional state of biomacromolecules that control cellular activity and perform metabolic work, such as intra- and inter-cellular signaling (e.g., via receptors and membrane transporters), metabolism (e.g., via enzymes), and the production of those biomacromolecules (e.g., via response elements on DNA), to list just a few of many examples.

Typically, only certain hormones fit a particular class of hormone receptors with sufficient complementarity to produce receptor-mediated effects (Chedrese and Celuch 2009). However, it would be a mistake to assume that hormones are the only endogenous chemicals that interact with hormone receptors. Hormones within related classes are usually derived from common precursors and share similar chemical structures, but structural similarities extend to many common endogenous molecules, including hormone precursors and hormone metabolites and intermediates and also products of various biochemical pathways (Chedrese and Celuch 2009), many of which have measurable affinity. Some of these also have efficacy for various types of hormone receptors, but those with no efficacy can still influence the system as antagonists. The total fraction of any receptor type that is occupied by ligands depends on the number of ligands present, their affinities for the receptor, and their concentrations in the compartments where the receptors reside. Thus, for example, potential exogenous ligands for a receptor such as ERα must compete for access to the receptor not only with the endogenous hormone E2, but with all naturally-occurring endogenous molecules that have affinity for ERα, of which there are many (Baker 2013).

The endogenous milieu is formidable. Using the estrogenic pathway as an example again, 27-hydroxycholesterol (27-HC), dehydroepiandrosterone (DHEA) and its metabolites DHEA-sulfate, androstenedione and androstenediol are endogenous, naturally occurring products of human metabolism that all exhibit measurable affinity and efficacy for both ERα and/or ERβ. Androstenediol is the most potent of these at ERα (Miller et al. 2013), exhibiting full agonist activity in vitro, i.e., its maximal activation of ERα is equivalent to that of the endogenous hormone E2. Androstenediol has approximately 1/100th the affinity of E2 (Miller et al. 2013) and thus displaces a quantifiable fraction of the endogenous estrogen E2 from ERα in vitro when its concentrations are within 100-fold that of the natural hormone. Since androstenediol circulates in blood at low (nM) concentrations compared with low (pM) concentrations of E2, it occupies a significant fraction of ERα throughout the body even though its affinity for ERα is 100-fold lower than that of E2.

As well, 27-hydroxycholesterol, a precursor of DHEA, binds to ERα (He and Nelson 2017), albeit with low affinity (i.e., < 1/1000th that of E2) but circulates in blood at concentrations of 150–730 nM. Thus, at normal circulating concentrations, 27-hydroxycholesterol will also occupy a functionally significant fraction of ERα in the body. Dozens of endogenous non-estradiol ERα ligands circulate in the bloodstream, but the DHEA derivatives alone demonstrate that to act via estrogen receptors or to alter the endogenous estrogenic tone, exogenous chemicals must compete not only with estradiol, but also with an overwhelming number and concentration of other natural endogenous ligands, some of which are sufficiently potent to exert cellular effects individually. Without sufficient affinity to compete against the endogenous milieu and occupy a functionally significant fraction of a particular class of hormone receptors, exogenous chemicals or mixtures of chemicals have no potential to alter the functional status of those receptors or their signaling pathways in hormone-sensitive cells and tissues, and thus, pose no endocrine disruptive hazard. We view the naturally occurring endogenous metabolic milieu as a damper, or buffer, that prevents weak receptor ligands from exerting physiological effects. As a specific example, it seems likely to us that the endogenous milieu of ERα ligands can explain the physiological basis of our observed HRPT of 1 × 10^–04^ relative to E2.

## Methodology

A simple equation derived by Gaddum in 1937 (Eq. 3) describes equilibrium receptor occupancy by ligand A in the presence of ligand B (Rang 2006; Colquhoun 2006).

3 $$P_A=\frac{C_A}{1+ C_A + C_B}$$

*P*_A_ is the equilibrium combined receptor occupancy, *C*_A_ is the normalized concentration of *A*, *C*_A_ = [*A*]/*K*_A_, with *K*_A_ being the equilibrium dissociation constant for A and, similarly, *C*_B_ = [*B*]/*K*_B_ (Colquhoun 2006).

Polynomial expansion of this equation enables calculating the fraction of receptors occupied by any individual ligand in the presence of *n* competing ligands of varying affinity. These equations are well accepted in endocrine pharmacology, are validated for clinical relevance, and have been available for decades as computerized algorithms. In the physiological state, hormone receptors are bound in continuous equilibrium with the primary endogenous ligand and the endogenous metabolic milieu, therefore, the Gaddum equilibrium binding equation is appropriate for testing our general hypothesis with the specific case of ERα occupancy.

We used the Gaddum equation in a series of receptor occupancy calculations to test the specific hypothesis that high blood concentrations of a chemical with potency equal to the HRPT (Borgert et al. 2018) could attain sufficient occupancy of ERα receptors to alter the estrogenic tone of an intact animal amidst normal levels of even a subset of endogenous natural ERα-ligands. The subset of endogenous ERα ligands used for these calculations includes DHEA, DHEA-sulfate, androstenediol, E2, estrone (E1) and estriol (E3). To establish the physiologically successful level of receptor occupancy, receptor occupancy was calculated for E2 amidst the normal ranges of the other endogenous natural ERα-ligands, DHEA, DHEA-sulfate, androstenediol, E1 and E3. These calculations provide a conservative estimate of the potential for an exogenous chemical to alter the estrogenic tone of an intact animal by virtue of the fact that the true level of ERα occupancy by the entire endogenous metabolic milieu would be even higher than for only these five endogenous ligands.

Affinity values for the endogenous ERα ligands androstenediol, DHEA, DHEA-sulfate, E1, E2, E3, and their circulating concentrations in humans were determined from the published literature. An affinity of 1 × 10^–4^ relative to that of E2 was used to simulate an exogenous ERα ligand with potency equal to the HRPT proposed by Borgert et al. (2018), and *K*_d_ values 10 and 100-fold lower were used to simulate exogenous ligands with potencies above the HRPT. Where only rat plasma levels were available, a concentration of tenfold that in rat was assumed for humans based on published information (Nilsson et al. 2015; Witorsch 2002). Table 1 lists the ligand affinities expressed as *K*_d_, and the median and minimum plasma concentrations used in receptor occupancy calculations for each endogenous steroid and for theoretical exogenous ligands with potency at, and above, the previously proposed HRPT (Borgert et al. 2018).

**Table 1:** Dissociation constants and plasma concentrations used for receptor occupancy calculations

Ligand label	Endogenous steroid or exogenous ligand	Kd (M)	Affinity relative to E2	Mid-point plasma concentration (M)	Minimal plasma concentration (M)
A	17β-Estradiol (E2)	1.0E-10	1.00E + 00	1.84E-09	7.34E-12
B	Androstenediol	4.5E-09	2.22E-02	1.8E-09	1.1E-09
C	DHEA	1.1E-06	9.09E-05	1.9E-08	7.0E-09
D	DHEA-Sulfate	1.1E-04	9.09E-07	7.8E-06	5.4E-07
E	Estrone (E1)	2.2E-09	4.55E-02	1.5E-07	2.7E-11
F	Estriol (E3)	2.2E-09	4.55E-02	1.5E-07	2.7E-11
G	HRPT Ligand	1.0E-06	1.00E-04	1.0E-06	1.0E-09
H	Supra-HRPT Ligand	1.0E-07	1.00E-03	1.0E-06	1.0E-09
I-a	Supra-HRPT Ligand 10X	1.0E-08	1.00E-02	1.0E-06	1.0E-09
I-b	Supra-HRPT Ligand 10X	1.0E-07	1.00E-03	1.0E-05	1.0E-08

### Androstenediol (Adiol)

Affinity values (EC_50_, IC_50_, *K*_d_) for Adiol binding to ER have been published (Adams et al. 1981; Garcia and Rochefort 1979; Hackenberg et al. 1993; Kuiper et al. 1997; Poortman et al. 1975; Miller et al. 2013). For receptor occupancy calculations, we used the *K*_d_ of 4.5 nM reported by Garcia and Rochefort (1979). Endogenous levels in women have been reported to range from 1.1 nM post-menopause to 2.5 nM pre-menopause (Maroulis and Abraham 1976). For receptor occupancy calculations shown here, values of 1.1 nM and 1.8 nM were used to represent minimal and mid-point plasma concentrations.

### DHEA

DHEA has been reported to have affinity for ERα, with *K*_i_ values ranging from 245 nM (Kuiper et al. 1997) to 1.1 µM (Chen et al. 2005; Miller et al. 2013) and an EC_50_ value of 1 µM (Bruder et al. 1997). *K*_i_ values are the dissociation equilibrium constants for ligands that function as antagonists. DHEA possesses affinity for ERα approximately four orders of magnitude lower than E2 (Nephew et al. 1998). Plasma levels of 7–31 nM have been reported (Chen et al. 2005; Miller et al. 2013). For receptor occupancy calculations reported here, a *K*_d_ of 1.1 µM was used, and blood levels of 7 and 19 nM were used for minimal and mid-point plasma concentrations, respectively.

### DHEA-sulfate

Based on transcriptional activation of ERα in HepG2 and HEK-293 cells reported by Miller et al. (2013) and in MVLN cells reported by Bjerregaard-Olesen et al. (2016), DHEA-sulfate appears to have an EC_50_ value of approximately 20 µM, from which a *K*_d_ of 110 µM was estimated for use in the receptor occupancy calculations reported here. Human plasma levels are reported to range from 0.5 to 15 µM (Bjerregaard-Olesen et al. 2016; Chen et al. 2005). Minimal and mid-point plasma concentrations of 0.5 and 7.8 µM were assumed for the receptor occupancy evaluation conducted here.

### 17β-Estradiol (E2)

The EC_50_ value of E2 at ERα has been reported to range from 2.5 pM to 1 nM (Borgert et al. 2018), which should reflect the ligand affinity of an endogenous hormone with full efficacy. For these calculations, we used the *K*_d_ value reported by Kuiper et al. (1997) of approximately 0.1 nM. E2 is used as the reference estrogen and was assigned a relative potency value of 1.00.

Circulating concentrations of estradiol peak during the follicular phase of the menstrual cycle in post-pubertal women and have been reported to range from 734 pM to greater that 1836 pM (ada.com, 2023; Frederiksen et al. 2020; Ramesh et al. 2022), with levels fifty times higher during pregnancy (Dzaja et al. 2009; Salas et al. 2006; Zhang et al. 2017). For receptor occupancy calculations, a concentration of 1.84 nM was used as the mid-point plasma estradiol concentration for E2. Circulating concentrations of E2 appear to be lowest in pre-pubertal males (0.3–20 pM) and females (2–15.3 pM) (Ankarberg-Lindgren et al. 2018; Ikegami et al. 2001; Klein et al. 1994). A large range has been reported (Bay et al. 2004) and levels rise more than tenfold during puberty (Di Meo et al. 2023). For receptor occupancy calculations, 7.34 pM was used as a minimal plasma concentration of E2.

### Estrone (E1)

A *K*_d_ for E1 of 2.2 nM was estimated based on the *K*_i_ value published by Kuiper et al. (1997), consistent with a potency of approximately 4.55 × 10^–2^ relative to E2. E1 concentrations appear to range from 12 to 53 pM in young boys and 16–99 pM in young girls (Ankarberg-Lindgren et al. 2018) and from 155 pM (Nilsson et al. 2015) to 370 pM (Bjerregaard-Olesen et al. 2016) in premenopausal women. For receptor occupancy calculations conducted here, values of 27 pM and 0.15 µM were used as the minimal and mid-point plasma concentrations.

### Estriol (E3)

The affinity of E3 was assumed to be equal to that of E1, and thus, the same *K*_d_ value of 2.2 nM was used for E3, consistent with a potency of approximately 4.55 × 10^–2^ relative to E2. Minimal and mid-point plasma levels of E3 were also assumed to be similar to E1. Therefore, identical values were used for receptor occupancy calculations for both E1 and E3.

## Results and discussion

Table 2 lists the results of fractional receptor occupancy calculations for the primary endogenous estrogen 17β-estradiol (E2) alone and in the presence of five endogenous ERα ligands. As shown in Table 2, Row Q, E2 alone, in the absence of any competing ERα ligands, would occupy only about 7% of estrogen receptors at minimal plasma concentrations, similar to those that have been measured in pre-pubertal boys, but would nearly saturate estrogen receptors (~ 95%) at plasma concentrations similar to those observed in post-pubertal, non-pregnant women. It is important to appreciate that such calculations yield artificial, non-physiological representations of receptor occupancy because they consider only the endogenous primary hormone in the absence of competition by the endogenous metabolic milieu that is present naturally in the blood and bodily fluids. Similarly, most in vitro receptor binding and receptor transactivation assays used in toxicology to assess interaction with estrogen receptors do so in the absence of competing ligands, both exogenous (e.g., phenol red) and endogenous (e.g., components of serum). This is done to maximize sensitivity, but the results should be interpreted in light of the physiological-irrelevance of such conditions, as should receptor occupancy calculations that mimic them.

**Table 2:** Fractional receptor occupancy of sub-HRPT chemical in the presence of normal plasma concentrations of endogenous steroidal ERα ligands

Row	Ligand Mixture	Percent ER occupied at mid-point plasma concentration (M)	Percent ER occupied at minimal plasma concentration (M)
Q	Fraction occupied by E2 alone	94.8347	6.8381
R	Fraction occupied by E2 in presence of B – F	11.7533	5.4223
S	Fraction occupied by E2 in presence of B – F, G	11.6785	5.4183
T	Fraction occupied by E2 in presence of B – F, H	11.0461	5.3826
U	Fraction of ER occupied by A-F	99.3598	26.1263
V	Fraction of ER occupied by A – F, G	99.3639	26.1809
W	Fraction of ER occupied by A – F, H	99.3984	26.6681
X	Difference Due to G	0.0041	0.0545
Y	Difference Due to H	0.0385	0.5417
Z	Difference Due to I-a or I-b	0.2498	5.0819

Table 2, Row R shows the fraction of ERα occupied by E2 in the presence of five endogenous ERα-ligands, B–F, at mid-point and at minimal concentrations found in human blood. At minimal plasma concentrations of this subset of the endogenous metabolic milieu, the fractional receptor occupancy by E2 would be reduced only slightly—by about 1.4% (from 6.8381 to 5.4223%)—relative to the fraction occupied by E2 in the absence of this endogenous milieu (Row Q). This strongly suggests that a change in ERα-receptor occupancy of 1.4% is not relevant in humans, even at the most sensitive life-stage when E2 levels are lowest, and assuming that the endogenous metabolic milieu is also at its lowest. At mid-point plasma concentrations, the endogenous metabolic milieu is calculated to occupy a much greater fraction of ERα, and the fractional receptor occupancy by E2 would be reduced dramatically, from approximately 95% (94.8347%) for E2 alone (Row Q) to approximately 12% (11.733%) in the presence of the five additional endogenous ERα ligands (Row R).

Relevant to the hypotheses tested here, Row R shows that the normal range of ERα occupancy by E2 may be 5.4–11.7%. If one considers that a range of approximately 5.4–11.7% of ERα is occupied by E2 under normal physiological conditions, alterations of E2 receptor occupancy within this range may be considered unlikely to produce physiological effects and therefore, would be physiologically unable to alter endocrine function. A comparison of Rows R and Q shows the importance of considering the endogenous metabolic milieu when evaluating the potential for a chemical to produce physiologically relevant effects via a particular hormonal pathway. These calculations also indicate that the endogenous metabolic milieu is a primary determinant of ERα occupancy by the primary endogenous estrogen, E2. The endogenous milieu may be a more significant modulator of E2 activity during adult life stages when E2 levels are higher due to the concomitant higher concentrations of the other endogenous ERα ligands. Taken together, these calculations strongly suggest that to produce a physiologically relevant estrogenic effect, an alteration in E2 fractional ERα occupancy greater than 5% would be required.

The results of calculations shown in Table 2, Rows S and T are central to testing the hypotheses proposed herein. Table 2, Row S shows that when the receptor occupancy calculations include a hypothetical exogenous ERα ligand with affinity of 10^–4^ relative to E2, equal to the HRPT proposed previously (Borgert et al. 2018), the ERα occupancy due to E2 is essentially unaltered. This is regardless of whether the exogenous ligand is present at 1 nM amidst minimal plasma levels of the endogenous milieu (5.4223 to 5.4183% ≈ 0.004%) or at 1 µM amidst the mid-point plasma concentrations of the endogenous milieu (11.7533 to 11.6785% ≈ 0.075%). Row T shows that even if the exogenous ligand has affinity tenfold greater than the HRPT (10^–3^ relative to E2), no physiologically relevant change in receptor occupancy would be produced by this ligand at 1 µM amidst mid-point plasma levels of the five endogenous ligands (11.7533 to 11.0461% ≈ 0.7%), or at 1 nM amidst minimal endogenous ligand concentrations (5.4223 to 5.3826% ≈ 0.04%). These calculations provide compelling mechanistic support for the ERα-agonist HRPT proposed previously (Borgert et al. 2018) and the specific hypothesis tested here, based on the following considerations: (1) that exogenous ligands with affinity even tenfold greater than the HRPT (10^–4^ relative to E2) change E2 fractional receptor occupancy by less than 5%, a physiologically irrelevant change; (2) that the mid-point plasma concentration of the hypothetical exogenous ligand was assumed to be several orders of magnitude higher than is likely achievable for most exogenous chemicals (1 µM); and (3) that the minimal plasma concentration assumed (1 nM) for the exogenous ligand is also quite high, e.g., more than 200 times the maximum plasma concentration measured for one of the most well-studied exogenous chemicals, bisphenol A (BPA) (Mielke and Gundert-Remy 2009; Pande et al. 2019; Teeguarden and Hanson-Drury 2013; Teeguarden et al. 2013, 2016).

Calculations presented in Table 2, Rows U through Y allow a discussion of estrogenic activity in the broader context of total ERα occupancy by endogenous and hypothetical exogenous ligands and a more detailed consideration of the HRPT. Row U shows that the six endogenous ERα ligands considered here would occupy greater than 99% of ERα at normal concentrations measured in non-pregnant, post-pubertal women (“mid-point”), but only about 26% at the minimal plasma concentrations assumed to be present in pre-pubertal males. Row V shows that even amidst the minimal plasma concentrations of the endogenous milieu, a physiologically-relevant change in ERα occupancy would not be produced by introduction of an exogenous ERα ligand with affinity equal to HRPT potency (10^–4^ relative to E2), even if that ligand were present at a concentration of 1 nM (26.1809% versus 26.1263%). For comparison, 1 nM is more than 200-fold higher than the serum concentration measured for the most well-studied putative exogenous estrogen, BPA. Under those conditions, the exogenous ligand would contribute only 0.054% to the total ERα occupancy (Row X). Row W shows that no physiologically relevant change in ERα occupancy would be achieved even if the exogenous ligand had affinity tenfold higher than the HRPT potency amidst minimal concentrations of the endogenous milieu (26.6681% versus 26.1263%). Under those conditions, which assume a concentration of exogenous ligand likely unachievable in humans, the exogenous ligand would still contribute only 0.5417% (Row Y). Through similar comparisons, Rows V and W also show that amidst mid-point concentrations of the endogenous milieu, exogenous ligands with affinities equal to, or tenfold higher than the HRPT potency would produce no physiologically-relevant change in ERα occupancy when present at 1 µM, accounting for only 0.0041% and 0.0385% receptor occupancy, respectively.

Finally, the analysis was extended to ask for the potency or concentration at which an exogenous ligand would need to be present to overcome the potency threshold created by the endogenous metabolic milieu, even when present at only minimal concentrations. Row Z shows that to alter E2 receptor occupancy to a physiologically-relevant degree (> 5%), an exogenous ligand would need to have either an affinity approximately two orders of magnitude above the HRPT (10^–2^ relative to E2) if present in blood at 1 nM concentration, or, would need to achieve a concentration in blood of approximately 10 nM if its potency were only tenfold the HRPT (10^–3^ relative to E2).

It is important to appreciate that receptor occupancy calculations depend on both the potency and the circulating concentration of the ligand. Although it is sometimes assumed that any substance capable of interacting with a hormone receptor can produce an effect with sufficient exposure, this is clearly naïve. The receptor occupancy analysis shown here considered potential human exposures to exogenous substances but showed that the presence of a formidable endogenous metabolic milieu prevents the manifestation of estrogenic effects at concentrations physiologically achievable for most weak ERα ligands. This is evident from the earliest descriptions of reduced fertility in sheep. Although all sheep graze on plants that contain numerous botanical estrogens, reduced fertility occurred in those that grazed exclusively on a type of red clover that contains a high proportion of botanical estrogens with potencies above the HRPT of Borgert et al. (2018), such as Biochanin A, formononetic, coumestrol and genistein (Wyse et al. 2022). Thus, to be credible, hazard identification of EDCs should consider the potency of the substance at the molecular target and physiologically achievable concentrations of the substances (Autrup et al. 2015, 2020), and as demonstrated by the current analysis, whether physiologically achievable concentrations can overcome baseline occupancy of the molecular target by the endogenous metabolome.

**Fig. 1:**
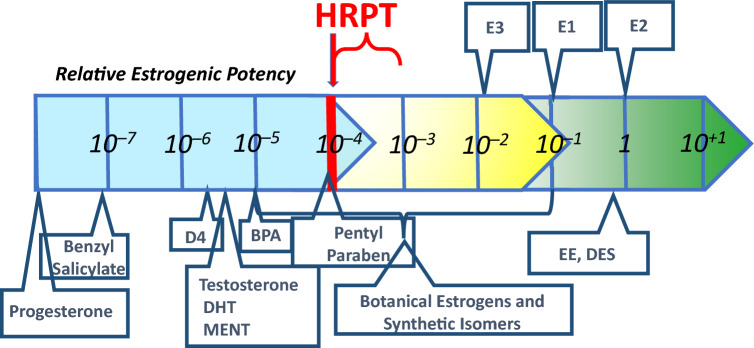
Revised Human-Relevant Potency-Threshold (HRPT) for the ERα-Agonist MoA. This Figure was adapted from Borgert et al. (2018) showing the conservatism in the threshold region beyond the originally proposed HRPT to as high as 10^–3^ relative to E2, in accordance with receptor occupancy calculations shown in Table 2. Also added to the original figure by Borgert et al. (2018) are potency estimates for three chemicals incorrectly alleged to be exogenous estrogens. Potency data for benzyl salicylate and BPA are from Natsch et al. (2021) and data for parabens are from Fayyaz et al. (2021)

The results shown in Table 2, Rows X, Y and Z have important implications for the ERα agonist HRPT originally proposed by Borgert et al. (2018). The results shown in Rows U–Z suggest that the HRPT estimate of 10^–4^ relative to E2 may be conservative by as much as tenfold, since ligands with affinities as high as 10^–3^ that of E2 would theoretically be unable to produce a physiologically relevant change in ERα occupancy, even when only five components of the endogenous metabolic milieu and the primary endogenous hormone, E2 are present. Figure 1 provides a revised version of Fig. 1 from Borgert et al. 2018, showing the potential range of conservatism in the threshold potency extending beyond the originally proposed HRPT to as high as 10^–3^ relative to E2. Figure 1 also adds potency estimates for three chemicals that have been incorrectly alleged to be exogenous estrogens. Potency data for benzyl salicylate and BPA (Natsch et al. 2021) and for parabens (Fayyaz et al. 2021) reveal that these chemicals lack the potency to disrupt the estrogen pathway as agonists or antagonists.

In addition to endogenous ERα ligands considered in the calculations shown here, the endogenous metabolome includes many other ligands that can interact with estrogen receptors, including ERα. For example, 27-hydroxycholesterol has been deemed a physiologically-active Selective Estrogen Response Modifier (SERM) in humans based on an IC_50_ value of 1 µM, an EC_50_ value of approximately 50 µM, and a plasma concentration 0.15–0.9 µM (DuSell et al. 2008; He and Nelson 2017). Androgens also bind estrogen receptors, albeit with low affinity. The presence of these additional endogenous chemicals that interact with the estrogen receptor strengthens the results and interpretations of receptor occupancy calculations shown here.

The analyses shown here explain three observations. First, they explain why extracts of low-affinity botanical estrogens failed to elicit measurable estrogenic effects in early clinical trials but showed weak clinical efficacy in more recent trials that used extracts standardized to a higher proportion of the most potent botanical estrogens (Borgert et al. 2018; Messina 2014). Borgert et al. (2018) relied on a comprehensive review of clinical trials with soy-based supplements (Messina 2014) that showed clinical outcome was dependent on the isoflavone profile of the product. Messina (2014) discussed studies showing that efficacy depended on meeting a threshold intake of genistein, and that an intake of at least 19 mg genistein per day could be predicted to show 60% efficacy in hot-flash reduction. Borgert et al. (2018) also relied on laboratory studies with soy extracts in which uterotrophic effects in Wistar rats were observable with administration of a 46% soy extract containing a high ratio of high-potency components (genistin, genistein and glycitein) compared to lower-potency components, but not with administration of an equal amount of a 51% soy extract that contained a low ratio of those high-potency components (de Lima Toccafondo Vieira et al. 2008). Thus, administration of low-potency isoflavones—those with potencies near the HRPT, such as daidzein—produced no uterotrophic effect, while administration of high-potency isoflavones—those with potencies above the HPRT, such as genistein—was uterotrophic. This indicates that efficacy is explained not primarily by exposure, but according to whether the extract contains components with sufficient potency to overcome the background receptor occupancy of the endogenous metabolic milieu.

The above explanation is consistent with our calculation that a functionally significant change in E2 receptor occupancy might be achieved by a ligand with relative potency of 1E-03 at a blood concentration of 1E-05 M (Table 1, Row Ib as shown by Table 2, Row Z). Results of several studies that reported on botanical estrogen levels in humans indicate that serum concentrations of botanical estrogens with potencies above the HRPT can, under some circumstances, reach concentrations that could alter the functional status of ER-mediated pathways. Although in healthy women age 25 years and older who were not taking medications or dietary supplements, mean genistein levels have been reported to be in the range of 25–300 nM (Arai et al. 2000; Mortensen et al. 2009; Palma-Duran et al. 2015), women enrolled in a cardiovascular survey, whose blood levels were validated against samples from women taking an isoflavone supplement in a clinical trial, had median serum genistein and equol levels in the range of 140 µM (median) – 340 µM (mean) (Barsky et al. 2021). Adult men taking an isoflavone formulation in a clinical trial reached mean serum levels about tenfold lower, in the range of 27 µM (Busby et al. 2002). Infants fed soy-based formulas have been reported to attain mean serum genistein concentrations in the low µM range (Cao et al. 2009; Mortensen et al. 2009; Ryowon et al. 2004; Setchell et al. 1997). Thus, humans can attain levels of genistein that might, in some individuals, produce clinically observable effects. As discussed above, this is consistent with results from more recent clinical trials of dietary supplements containing botanical estrogens, which show a low degree of efficacy (Messina 2014).

Second, the analysis of receptor occupancy shown here also explains why the male and female reproductive tract abnormalities caused by DES, including vaginal adenocarcinoma, exhibited a clear threshold (Borgert et al. 2012; Dietrich 2010; Golden et al. 1998; Hoover et al. 2011), even though during the era that DES was used during pregnancy, exposures to weakly estrogenic and anti-androgenic environmental contaminants, such as chlorinated pesticides, polychlorinated biphenyls and chlorinated dioxins were much higher than current exposures and blood levels (Dietrich 2010; Golden et al. 1998). As noted in the methodology section of this paper, circulating concentrations of estradiol peak during the follicular phase of the menstrual cycle in post-pubertal women and reach levels fifty times higher than the “mid-point” condition during pregnancy. In humans, the fetus is exposed to exogenous and endogenous substances via the maternal circulation. Because estrogen receptor occupancy by E2 would be highest during pregnancy due to approximately 50-fold higher circulating E2 concentrations compared to the “mid-point” condition we assessed, it should be obvious that an exogenous substance incapable of occupying a functionally significant fraction of receptors at the mid-point concentration would be even less able to affect receptor occupancy during pregnancy. This is corroborated by the tragic experience with DES, an ERα ligand with potency well above the HRPT that exerts agonist and antagonist effects in utero and produced reproductive tract abnormalities and cancer in offspring when high doses were administered to pregnant women during the first trimester of pregnancy. As explained previously (Borgert et al. 2012), if the endocrine system had already been “activated” above the threshold by endogenous estrogens, to which had been added exposure to environmental estrogenic EDCs, then any additional exposure to a strong estrogen such as DES should have produced observable effects. However, environmental exposures to ERα ligands with potencies below the HRPT were irrelevant. As shown here, this is explained by their inability to displace a functionally significant fraction of E2 and evidenced by the fact that only the highest-dose DES regimens produced reproductive abnormalities. As shown by receptor occupancy theory, instead of providing the basis for additive effects, the endogenous metabolic milieu provides a buffer of receptor occupancy that blunts the ability of weak ligands to alter the functional status of the system. Our quantitative explanations for those observations strengthen the conclusion that generalized additivity approaches are scientifically untenable for mixtures of putative endocrine disruptive chemicals (Borgert et al. 2012).

Third, the analysis presented here indicates that dismissing the role of potency in identifying chemicals with potential to elicit effects via endocrine mechanisms—i.e., potential endocrine disruptors—is based on a misunderstanding or mischaracterization of the components of potency. First, the receptor occupancy calculations shown here demonstrate that under physiological conditions, hormone receptors are in a state of continuous equilibrium binding due to the overwhelming concentrations of endogenous components of the metabolome that interact with hormone receptors. It is untenable to posit that a very small change in ligand binding can result in a physiologically-relevant change in receptor occupancy under non-equilibrium conditions because non-equilibrium conditions do not exist physiologically. Second, pharmacological potency is a function of both the affinity and efficacy of an interaction between a small molecule (e.g., receptor ligand) and a functional biological macromolecule (e.g., a receptor) (Borgert et al. 2013, 2018). Neither aspect can be dismissed. A ligand with affinity so low that it cannot substantially affect receptor occupancy cannot produce a physiological effect, irrespective of its efficacy at the receptor. This is mathematically obvious for receptor antagonists based on the calculations shown here for competition with the endogenous ligand 17β-estradiol.

Efficacy was not considered in the calculations shown here for the sake of simplicity, but a consideration of efficacy reinforces the HRPT concept and the physiological basis of thresholds for endocrine disruptive effects; efficacy works in concert with laws of mass action to dictate cellular and biochemical responses. Because of its low receptor occupancy, a low-affinity ligand is unlikely to produce a cellular response, even if the ligand possessed intrinsic activity equal to the primary endogenous hormone. Furthermore, efficacy is determined by the particular pattern of gene expression that each different ligand induces. This forms the fundamental basis of the selective estrogen response modifier (SERM) concept (Gronemeyer et al. 2004; Kuiper et al. 1999). Since each ligand may produce a unique conformational change in the receptor that produces a different cellular signal, simultaneous ER activation by different ligands—e.g., a weak partial agonist and a full agonist is unlikely to alter receptor signaling. Cellular responses typically require the simultaneous activation of several receptors per cell to elicit a cellular response (Borgert et al. 2013 and citations therein). Unless multiple receptors are activated simultaneously within a cell by ligands with the same or very similar intrinsic activity, cellular response is unlikely to be produced. Thus, although affinity considerations are sufficient to establish the molecular/biochemical basis of the HRPT concept, as shown here, it is further strengthened by an understanding of the dual role of efficacy with the laws of mass action.

Receptor occupancy calculations shown here demonstrate that low-potency ERα-ligands, defined as those having relative potencies at or below the HRPT for ERα-agonists, have an infinitesimally low probability of displacing a sufficient fraction of bound estradiol from estrogen receptors to elicit a physiological response or to alter the functional status of the system. This would hold not only for single chemicals but also for mixtures of low-potency chemicals. There are several reasons that mixtures of low-affinity ligands are likely to be incapable of altering the functional state of the estrogen pathway. Our receptor occupancy calculations indicate that such mixtures would displace less than 5% of bound estradiol from estrogen receptors, even under excessive exposure assumptions and importantly, when competition from the endogenous metabolic milieu is not ignored.

Thus, the work presented here also has important implications for the assumption that mixtures of exogenous chemicals act additively, conferring upon putative EDC mixtures the ability to be harmful even when concentrations of the individual chemicals are too low to cause effects on their own. It appears that those theories have been developed without consideration of the physiologically relevant mixture of chemicals that exist naturally. This includes not only hormones but also an overwhelming excess of endogenous metabolites with potency and/or affinity at hormone receptors greater than that of most putative EDCs. In light of the work presented here, it would seem prudent to reconsider the validity of such theories on mixtures, especially their recommended adoption for regulatory purposes by some governmental scientific bodies (NRC 2009).

The physiological and biochemical basis of the HRPT demonstrated here is corroborated by the work of Pande et al. (2019), who evaluated the individual and total estrogenic contribution of endogenous and exogenous estrogens measured in human serum. They developed a method that integrated approaches for measuring total hormone concentrations and calculated the bioavailability of hormones at concentrations found in serum. Similar to the approach taken herein, they used equations to resolve multiple equilibria between estrogenic ligands and receptors. They found that fractional receptor occupancy at ERα and ERβ was dominated by E1, E2 and E3, as was the total estrogenic response. This included ligand specific differences in recruitment of co-activator proteins (RCA), which further corroborates our assertion that the biochemical and physiological basis underlying potency thresholds extends beyond receptor-mediated mechanisms. Receptor occupancy by BPA—a chemical with potency below the HRPT—was at least five orders of magnitude lower than E1, E2 or E3, and three orders of magnitude lower than the fetal derived E4, genistein, or daidzein, contributing less than 1/1000th of the normal daily variability in total serum estrogenicity in a cohort of pregnant women.

## Conclusions

Several conclusions derive from this work. The HRPT for ERα agonism (10^–4^ relative to E2) originally proposed by Borgert et al. (2018) is indeed conservative. Based on the work shown here, a potency within the range of 10^–4^ relative to E2 and below should be considered strong evidence against the potential for endocrine disruption of the estrogenic pathway. Second, even for chemicals with potency within 10^–3^ of E2, the potential for estrogenic endocrine disruption must be considered equivocal and subject to the presence of corroborative evidence. Third, and most importantly, this work demonstrates that the endogenous metabolic milieu is responsible for the observed ERα agonist HRPT, that the HRPT applies equally to receptor antagonists, and it provides a compelling mechanistic explanation for the HRPT that is grounded in basic principles of molecular kinetics using well characterized properties and concentrations of endogenous components of normal metabolism. The physiological and biochemical basis of the HRPT demonstrated here and the fact that its theoretic basis extends beyond receptor-mediated mechanisms is corroborated by the recent work of Pande et al. (2019).

## Abbreviations

Adiol: Androstenediol
BPA: Bisphenol A
DHEA: Dehydroepiandrosterone
DES: Diethylstilbestrol
*K*_d_: Equilibrium dissociation constant
EDCs: Endocrine-disrupting chemicals
E1: Estrone
E2: 17β-Estradiol
E3: Estriol
ERα: Estrogen receptor-alpha subtype
ERβ: Estrogen receptor-beta subtype
EC50: Half maximal effective concentration
IC50: Half maximal inhibitory concentration
HRPT: Human-Relevant Potency Threshold
K_i_: Equilibrium Inhibitory constant
µM: Micromolar
nM: Nanomolar
pM: Picomolar
SERM: Selective Estrogen Response Modifier
